# A genome-wide association study on growth traits of Korean commercial pig breeds using Bayesian methods

**DOI:** 10.5713/ab.23.0443

**Published:** 2024-04-15

**Authors:** Jong Hyun Jung, Sang Min Lee, Sang-Hyon Oh

**Affiliations:** 1 Jung P&C Institute, Yongin 16950, Korea; 2 National Institute of Animal Science, RDA, Cheonan, 31000, Korea; 3 Division of Animal Science, Gyeongsang National University, Jinju 52725, Korea

**Keywords:** Genome-wide Association Study, Growth Trait, Pig, Single Nucleotide Polymorphism

## Abstract

**Objective:**

This study aims to identify the significant regions and candidate genes of growth-related traits (adjusted backfat thickness [ABF], average daily gain [ADG], and days to 90 kg [DAYS90]) in Korean commercial GGP pig (Duroc, Landrace, and Yorkshire) populations.

**Methods:**

A genome-wide association study (GWAS) was performed using single-nucleotide polymorphism (SNP) markers for imputation to Illumina PorcineSNP60. The BayesB method was applied to calculate thresholds for the significance of SNP markers. The identified windows were considered significant if they explained ≥1% genetic variance.

**Results:**

A total of 28 window regions were related to genetic growth effects. Bayesian GWAS revealed 28 significant genetic regions including 52 informative SNPs associated with growth traits (ABF, ADG, DAYS90) in Duroc, Landrace, and Yorkshire pigs, with genetic variance ranging from 1.00% to 5.46%. Additionally, 14 candidate genes with previous functional validation were identified for these traits.

**Conclusion:**

The identified SNPs within these regions hold potential value for future marker-assisted or genomic selection in pig breeding programs. Consequently, they contribute to an improved understanding of genetic architecture and our ability to genetically enhance pigs. SNPs within the identified regions could prove valuable for future marker-assisted or genomic selection in pig breeding programs.

## INTRODUCTION

The pig breeding industry is comprised in several layers [1]. Due to these structural characteristics of the industry, the maternal and paternal lines are separated and selected, respectively, maximizing hybrid vigor in the crosses. In addition, since breeding pigs are maintained through generations of candidate selection by performance tests, the genes of the selected individuals are transferred to the lower layer and used to produce excellent finishers [2]. Therefore, the performance of breeding pigs is a factor that determines competitiveness in the pig industry. Since it takes a long time to move traits from purebreds to finishers, the ripple effect on the improvement of excellent breeding pigs is very large. In domestic Korean GGP farms, the main breeds used are the Yorkshire, Landrace, and Duroc purebreds.

In pig breeding programs, growth traits are economically important indicators of pig production performance and affect farm profits. Adjusted backfat thickness (ABF), average daily gain (ADG), and days to 90 kg body weight (DAYS90) are vital growth-related traits that are used to measure growth rate because of their significant impact on production efficiency [3]. Effectively managing and improving these traits can lead to higher production efficiency, better profitability, and a more sustainable and competitive pig farming operation. Pigs that grow faster with optimal backfat thickness and reach market weight more quickly are cost-effective for producers. Therefore, improvements in growth traits are necessary in the pig industry.

With the development of DNA technology that incorporates statistical methodology, it has become possible to study quantitative trait loci (QTL) or candidate genes [4,5]. Genome-wide association studies (GWAS) are a powerful approach in genetics and genomics to identify genetic variants (single nucleotide polymorphisms [SNPs]) associated with specific phenotypic traits or diseases, and are widely used to detect effective causal alterations and define narrow genomic regions in contact with these alterations [6]. In association analysis, DNA markers related to major genes can be identified because a SNP chip that covers the entire genome is densely integrated with SNP markers.

Bayesian methods in GWAS are invaluable when dealing with a large number of SNPs and a relatively small number of individuals [7]. In addition, by specifying an appropriate prior distribution for a SNP effect, only the effect affecting the phenotype is fitted to the model, and a small SNP effect is assumed to be zero depending on its π-value (a measure of statistical significance). By setting a threshold for SNP effects, the number of false positives during analysis can be controlled. SNPs with small effects that do not pass this threshold may be considered as not having a significant impact on the phenotype. Therefore, it is advantageous to find the candidate region for GWAS because the inferences are based on joint posterior distribution, which accounts for all unknown parameters [8,9]. Furthermore, it is possible to increase the efficiency of genetic improvement in livestock by increasing the accuracy of genetic ability evaluation through additional information on DNA markers with significant effects discovered during GWAS [10].

The aim of this study was to identify significant regions and candidate genes related to ABF, ADG, and DAYS90 in commercial Korean GGP pig (Duroc, Landrace, and Yorkshire) populations. This was achieved by conducting a GWAS using SNP chip platforms and the Bayesian method (BayesB with response variable).

## MATERIALS AND METHODS

### Phenotypic data

In this study, phenotypic data were collected from purebred Duroc (38,941), Landrace (23,451), and Yorkshire (91,146) pigs raised at a GGP farm in Korea between 2005 and 2022. The ADG was obtained from the difference in final weight and initial weight divided by the number of feed intake days. The BFAT was calculated based on the average fat thickness values of the shoulder (on the fourth thoracic vertebrae), mid-back (on the last thoracic vertebrae), and loin (on the last lumbar vertebrae) measured using an amplitude mode (A-mode) ultrasound device (PIGLOG 105). The DAYS90 was estimated according to the suggestions of the Korean Swine Performance Recording Standards (KSPRS), following previously reported procedures [3].

### Genotypic data

Genomic data were collected using the Affymetrix Axiom 53K, Affymetrix Axiom 650K (Affymetrix Inc., Santa Clara, CA, USA), and Illumina Porcine SNP60K v2 (Illumina, Inc., San Diego, CA, USA) platforms.

A total of 5,359 pigs were included in the analysis. Specifically, 1,029 Duroc pigs were genotyped using Illumina60Kv2 (818 pigs) and Axiom650K (211 pigs), 472 Landrace pigs were genotyped using Axiom650K (143 pigs) and Illumina60Kv2 (329 pigs), and 3,858 Yorkshire pigs were genotyped using Axiom53K (479 pigs), Illumina60Kv2 (2,932 pigs), and Axiom650k (447 pigs).

SNP markers without map information, those existing on sex chromosomes, and those with a call rate of 0.95 or less and genomic data that overlapped were excluded from the analysis. In total, 43,861, 52,580, and 52,403 SNPs from Duroc, Landrace, and Yorkshire pigs, respectively, were used for the analysis. The collected genomic data were analyzed by imputation with a medium-density (MD) platform (Illumina Porcine SNP60Kv2) using FImuteV3 [11] for each breed (Table 1). Since the FImputeV3 program does not provide an r-square value, the imputation accuracy was estimated using leave-one-out cross validation.

### Statistical methods

#### Estimation of genetic parameters

To estimate the genetic parameters, a linear animal model including additive genetic effects and fixed effects (sex, contemporary group) was applied to the multivariate model. The likelihood function logarithm (**log_e_ L**) was used to determine the most appropriate models for the trait. The analysis was performed using the ASREML4.1 program [12], and the analysis model was as follows:


$$y=\mathrm{Xb}+Z_{a}u_{a}+e$$

where **y** is the vector of observation (ABF, ADG, DAYS90), **X** and **Z_a_** are the incidence matrices for fixed and random effects, **u_a_** is the vector of the additive genetic effect, and **e** is the vector of the residual effect.

#### Response variables

Response variables for genomic analysis were estimated using estimated genetic parameters, variance components, estimated breeding values (EBV), and reliability according to the model for the genetic evaluation of each trait. Genomic analysis typically involves estimating response variables for specific traits. In this case, the response variables are related to growth traits such as ABF, ADG, and DAYS90. The estimates of genetic and residual variances and heritability for these traits are shown in Table 2.

Deregressed estimated breeding values (DEBV) including parent average were re-estimated using the EBV and reliabilities of each individual to help provide more accurate and reliable estimates of an individual’s genetic merit, particularly in situations where data is limited or unreliable. In addition, the weighting factor was calculated using the following formula [10]:


$$\omega_{i}=\frac{\left(1-h^{2}\right)}{\{c+\left[\frac{1-r_{i}^{2}}{r_{i}^{2}}\right]\}h^{2}}$$

where $r_{i}^{2}$ is the reliability of EBVs, *h*^2^ is the heritability of each trait, *c* is the genetic variance ratio explained by the SNP marker information assumed to be 0.4 [13]. The weighting factor was then applied to the GWAS to explain the heterogeneous variance resulting from the different reliabilities of each individual from the re-estimated response variables.

Once the response variables were converted into DEBVs, GWAS was performed after removing individuals with a reliability (reliability EBVs – reliability of parent average) of 0.1 or less.

#### Bayesian Method for GWAS

To estimate the effects of SNP markers for GWAS analysis, BayesB with a fixed π value of 0.99 was used [14]. This analysis usually employs a high-density marker dataset which contains an extensive number of genetic loci as well as multiple samples for each locus. Therefore, a more targeted analysis was performed by focusing on identifying candidate gene regions. Parameters and effects were obtained using Gibbs sampling for a total number of 50,000 Markov chain Monte Carlo (MCMC) iterations and a sampling interval (thinning) of 5. The first 5,000 MCMC iterations were discarded for burn-in before estimating posterior means of marker effects and variances. As autocorrelation occurs within the Markov chain, it was carried out to prevent the occurrence of bias in advance, and the response variable was estimated as follows:


$$y_{i}=\mu +\sum_{j=1}^{k}Z_{ij}u_{j}\delta_{j}+e_{i}$$

where *y_i_* is the response variable (DEBVs or EBVs), *μ* is the population mean, *k* is the number of markers, *Z_ij_* is the allelic state (0, 1, 2) at marker *j* in individual *i*, *u_j_* is the random substitution effect for marker *j*, and *δ_j_* indicates the presence or absence of a marker in the model (0 or 1).

In the case of the threshold, to search for a significant 1 Mb region through GWAS analysis, a region with ≥1% additive genetic variance explanatory power among the total genetic variance that can be explained by SNP markers is defined as a region with a significant effect. The model frequency statistic was estimated and used to select SNP markers with significant effects on each trait. A total of 2,451 1 Mb regions were included in the total region, and the additive dielectric dispersion ratio of each region was approximately 0.041% (100%/2,451).

The BayesB method uses the *t*-distribution as a prior distribution for SNP marker effects, and is sampled from the prior assumption that each SNP marker has a different variance. This process was performed using the GenSel4R program [15].

## RESULTS AND DISCUSSION

The average ADG for Duroc, Landrace, and Yorkshire pigs was 675±72 g, 646±66 g, and 612±75 g, the ABF was 12.83± 2.40 mm, 12.83±2.60 mm, and 13.73±2.97 mm, and the DAYS90 was 136.7±11.2 days, 143.7±11.6 days, and 148.8± 14.7 days, respectively (Table 3). GWAS was performed using SNP markers imputed to the Illumina PorcineSNP60. Bayesian GWAS applies a threshold for the significance of SNP markers based on significant windows (≥1% genetic variance). A single QTL can affect multiple SNPs because of the high linkage disequilibrium within adjacent SNPs [16].

**Figure 1:**
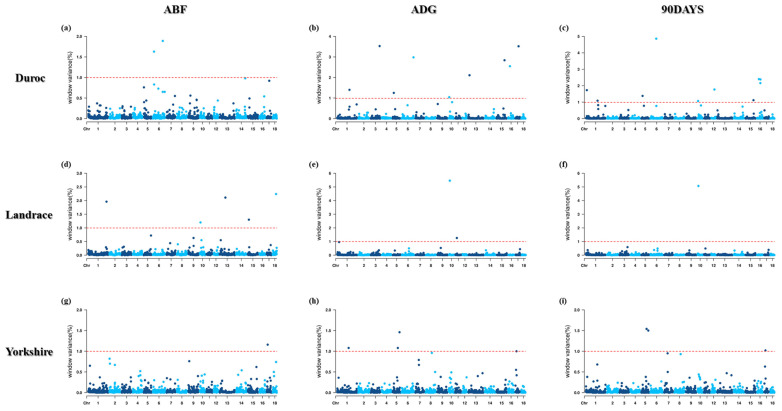
Manhattan plots for genome-wide association study (GWAS) based on Bayesian B (BayesB) methods of adjusted backfat thickness (ABF), average dairy gain (ADG), days to 90 (90DAYS) by pig breeds.

Therefore, to identify the significant 1 Mb windows, including SNPs, GWAS was performed with three traits (ABF, ADG, and DAYS90) based on BayesB. A total of 28 window regions were found, including 52 informative SNPs based on their genetic effects (Tables 3 to 5; Figure 1).

### Adjusted backfat thickness

Analysis found eight significant windows (≥1% genetic variance) with 18 informative SNPs. For ABF, 16 genes were located on *Sus Scrofa* chromosome (SSC) 6 in Duroc pigs, SSCs 1, 10, 13, 15, and 18 in Landrace pigs, and SSC17 in Yorkshire pigs (Table 4). These regions explained 1.16% to 2.24% of the total genetic variance for ABF, and 17 genes were annotated in these genomic regions.

In Landrace pigs, the most significant 1 Mb window region, explaining 2.24% of the additive genetic variance, was captured on SSC18 at 46 Mb (H3GA0051040). In Duroc pigs, the most significant window, explaining 1.84% of the additive genetic variance, was captured on SSC6 at 138 Mb, and included six SNPs (MARC0037040, M1GA0008914, DRGA 0006864, ASGA0103810, ALGA0113757, and ALGA0036942). In Yorkshire pigs, the most significant window explained 1.16% of the additive genetic variance and was captured on SSC17 at 13 Mb (ASGA0089601).

The *NFE2L3* gene adjacent to H3GA0051040, which was the most significant region in Landrace pigs, is required for myoblast differentiation and fusion in cellular processes, autophagy, and endoplasmic reticulum [17], and it is also associated with lipids in humans [18] and pigs [19].

The *MRPS22* gene, which located on SSC13 at the 80 Mb region, impairs mitochondrial mRNA translation and lowers coupling efficiency and energy storage, thereby altering the energy balance with potential consequences on lipid accumulation and adiposity [20]. Moreover, it has been suggested as a new susceptibility gene for human obesity [21,22].

All regions associated with ABF in Duroc pigs were found on SSC6. Although there have been no studies related to ABF, many studies have searched for the QTL in this SSC6 region associated with ABF [23–25]. In Landrace pigs, the *SLC23A2* gene on SSC17 at the 13 Mb region encodes sodium-coupled vitamin C transporter 2 (SVCT2), whose expression may be regulated by insulin-like growth factor signaling [26]. Similarly, its association with ABF has not been studied. Thus, research using a high-density genotyping chip is needed to identify the region.

### Average daily gain

For ADG, 25 informative SNPs were identified in 10 significant window regions (≥1% genetic variance) (Table 5). These regions explained 1.00% to 5.46% of the total genetic variance for ADG, and 31 genes were annotated in these genomic regions. Candidate regions associated with ADG were identified in SSCs 1, 3, 5, 6, 10, 13, 15, and 17 in Duroc pigs, SSCs 10 and 11 in Landrace pigs, and SSCs 1, 5, and 17 in Yorkshire pigs.

In Landrace pigs, the most significant 1 Mb window region explained 5.46% of the additive genetic variance and was captured on SSC10 at the 11 Mb region (DRGA0010301). In Duroc pigs, the most significant window explained 3.53% of the genetic variance and was captured on SSC3 at the 123 Mb region (MARC0091117). In Yorkshire pigs, the most significant window explained 1.46% of the genetic variance and was captured on SSC5 at the 95 Mb region (ASGA0026863).

The *FAM177B* gene adjacent to SSC10 at the 11 Mb region, which was the region most significantly related to ADG, is related to body size in Hulun Buir sheep [27]. The *TRIB2* gene, which is close to SSC3 at the 123 Mb region, the most significant region in Duroc pigs, is related to growth traits in Thoroughbred pigs [28]. The *RBMS3* gene, which is close to SSC13 at the 15 Mb region, is related to growth traits in cattle [29].

In Yorkshire pigs, the *MGAT4C* gene close to SSC5 at the 95 Mb region coincided with the region discovered in a study on Nero Siciliano pigs [30], which is also consistent with the results of a study on Italian Large White pigs [31].

The *RNF152* gene adjacent to SSC1 at the 159 Mb region coincided with the region discovered in a study on ADG in Landrace×Large Whites [32] and that discovered in a study on ABF and DAYS100 in Duroc pigs. Lee et al [16] examined ABF, DAYS90, loin muscle area, and lean percentage in Duroc pigs.

The *FERMT1* gene adjacent to SSC17 at the 15 Mb region positively regulates the transforming growth factor beta (TGF-beta) receptor signaling pathway in pigs and is significantly associated with carcass length. The *BMP2* gene is involved in the TGF-beta signaling pathway, playing a role in bone and cartilage development, and has been proposed as a strong candidate gene for carcass length [33]. In addition, it has been associated with body weight and body conformation traits in pigs [27].

### Days to 90 kg

For DAYS90, 25 SNPs were identified in eight significant windows (≥1% genetic variance) (Table 6). The candidate regions associated with DAYS90 were identified at SSCs 1, 5, 6, 10, 12, 15, and 16 in Duroc pigs, SSC10 in Landrace pigs, and SSCs 5 and 17 in Yorkshire pigs. These regions explained 1.02% to 5.07% of the total genetic variance, and 28 genes were annotated in these genomic regions.

In Landrace pigs, the most significant 1 Mb window region explained 5.07% of the additive genetic variance and was captured on SSC10 at the 11 Mb region (DRGA0010301). In Duroc pigs, the most significant region, explaining 4.86% of the variance, was captured on SSC6 at the 80 Mb region (H3GA0018314). In Yorkshire pigs, the most significant region explained 1.54% and was captured on SSC5 at the 71 Mb region (INRA0019895, ASGA0026241, ASGA0026236, and ALGA0032782).

The *LACTBL1* gene is located on SSC6 at the 80 Mb region, the most significant region in Duroc pigs, and has been associated with weight among the British population [34].

The *FAIM2* gene, which is located on SSC5 at the 15 Mb region, is correlated with muscle mass in cattle and it is a candidate gene for growth and carcass traits [35]. It is also closely related to obesity in humans, and many studies have been conducted with the *MC4R* gene [36,37]. NCKAP5L, located at the same position, has also been reported as a candidate gene for daily weight gain, which is a growth trait in cattle [38,39].

PIK3R1, located on SSC6 at the 45 Mb region, is directly related to lipid metabolism [40] and is involved in skeletal muscle differentiation and proliferation [41]. In addition, Chen et al [42] reported that it regulates feed intake and fat deposition in Chinese Laiwu pigs, while another study reported a QTL closely related to backfat thickness in pigs [43,44].

For the identified significant regions, there were six overlapping windows for ADG and DAYS90, which explained the different proportions of genetic variance in these two traits. For complex quantitative traits, it was assumed that the linear effects of genes fit the average of the traits completely. However, in practice, the effects of genes are not always linear for traits, and the nonlinear assumption is more appropriate, which means that genes contribute differently and QTL has pleiotropic effects between traits. The region with the largest explained genetic variance for ADG and DAYS90, located on SSC10 at the 57 Mb region, seemingly had pleiotropic effects on growth traits in pigs.

## CONCLUSION

We identified 28 significant window regions associated with three growth traits (ABF, ADG, and DAYS90) in Duroc, Landrace, and Yorkshire pig populations using the Bayesian GWAS method. The genetic variance of the identified windows varied from 1.00% to 5.46%.

Furthermore, 14 genes with related functional validation in previous studies were identified as candidate genes for growth traits. Such a full use of phenotypic and genotypic data and genealogical information will further advance our understanding of genetic architecture and accelerate the genetic improvement of economically important traits in pigs. In addition, the SNPs within the identified regions may be useful for marker-assisted selection or genomic selection in future pig breeding programs.

## Figures and Tables

**Table 1:** Basic statistics of single-nucleotide polymorphism data set

Breed	Description	Commercial genotyping platforms		

		Axiom 53K	Axiom 650K	Illumina 60Kv2
Duroc	Total number of animals	-	211	818
	No. duplicated animals	-	11	25
	No. target animals	-	200	764
	No. reference animals	-	-	159
	No. genotyped animals after imputation	-	-	923
	Total number of SNPs	-	658,692	61,565
	No. markers on autosome	-	592,052	58,863
	No, Selected markers after QC	-	592,024	58,845
	No. markers for analysis	-	-	43,861
Landrace	Total no. of animals	-	143	329
	No. duplicated animals	-	11	28
	No. reference animals	-	132	301
	No. target animals	-	-	132
	No. genotyped animals after imputation	-	-	433
	Total no. of SNPs	-	658,692	61,565
	Markers on autosome	-	592,052	58,863
	Selected markers after QC	-	592,024	58,845
	No. markers for analysis	-	-	52,580
Yorkshire	Total no. of animals	479	393	2,932
	No. duplicated animals	50	30	301
	No. reference animals	429	364	2,631
	No. target animals	-	-	2,631
	No. genotyped animals after imputation	-	-	3,424
	Total no. of SNPs	55,374	658,692	61,565
	Markers on autosome	49,732	592,052	58,863
	Selected markers after QC	49,624	592,024	58,845
	No. markers for analysis	-	-	52,403

SNP, single-nucleotide polymorphism; QC, quality control.

**Table 2:** Variance components and heritabilities for adjusted backfat thickness, average dairy gain, days to 90 kg by pig breed

Breeds	Trait	Genetic variance	Residual variance	Phenotypic variance	Heritability
Duroc	ADG	0.12	0.30	0.42	0.28
	ABF	1.55	2.88	4..43	0.35
	DAYS90	0.29	0.68	0.97	0.30
Landrace	ADG	0.12	0.22	0.34	0.35
	ABF	2.24	2.88	5.12	0.44
	DAYS90	0.36	0.60	0.96	0.37
Yorkshire	ADG	0.12	0.23	0.35	0.33
	ABF	3.04	3.97	7.01	0.43
	DAYS90	0.39	0.83	1.22	0.32

ABF, adjusted backfat thickness; ADG, average daily gain; DAYS90, days to 90 kg.

**Table 3:** Frequencies, means and standard deviations for adjusted backfat thickness, average dairy gain, days to 90 kg by pig breed

Breeds	Sex	N	ABF (mm)	ADG (g)	DAYS90 (d)
Duroc	Boar	17,523	12.74±2.02	684.74±70.29	134.05±10.38
	Gilt	21,418	12.86±2.56	670.61±73.85	138.64±11.18
	Total	38,941	12.83±2.40	675.01±72.21	136.71±11.21
Landrace	Boar	10,084	12.71±2.73	643.74±60.56	140.35±10.42
	Gilt	13,367	12.93±2.42	648.59±64.29	145.55±12.29
	Total	23,451	12.83±2.60	646.21±66.22	143.74±11.62
Yorkshire	Boar	36,458	13.55±2.54	608.74±71.10	145.64±13.58
	Gilt	54,688	13.97±2.48	620.49±70.97	149.77±13.96
	Total	91,146	13.73±2.97	612.13±75.10	148.82±14.73

ABF, adjusted backfat thickness; ADG, average daily gain; DAYS90, days to 90 kg.

**Table 4:** Summary of informative single-nucleotide polymorphisms in the significant 1-Mb windows for adjusted backfat thickness by breed

Breed^1)^	SSC_Mb	GV (%)	Informative SNP	Position	Effect	Model frequency	Region annotation	Gene annotation
DD	6_138	1.89	MARC0037040	138.70	0.055	0.132	Intergenic	*CRYZ* (dist = 11,423)
								*FPGT* (dist = 470,843)
			M1GA0008914	138.50	0.070	0.161	Intergenic	*LHX8* (dist = 152,772)
								*TYW3* (dist = 163,579)
			DRGA0006864	138.20	−0.030	0.081	Intergenic	*LHX8* (dist = 222,275)
								*TYW3* (dist = 94,076)
			ASGA0103810	138.00	0.018	0.057	Intergenic	*LHX8* (dist = 341)
								*TYW3* (dist = 316,010)
			ALGA0113757	138.60	−0.021	0.064	Intergenic	*CRYZ* (dist = 299,075)
								*FPGT* (dist = 183,191)
			ALGA0036942	138.90	0.029	0.079	Intergenic	*CRYZ* (dist = 36,545)
								*FPGT* (dist = 445,721)
	6_16	1.63	MARC0033972	16.80	0.109	0.260	Intergenic	*U6* (dist = 635,716)
								*PSMD7* (dist = 58,944)
			MARC0029800	16.40	0.053	0.142	Intergenic	*U6* (dist = 592,247)
								*PSMD7* (dist = 102,413)
			ALGA0034650	16.30	0.063	0.159	Intergenic	*U6* (dist = 482,216)
								*PSMD7* (dist = 212,444)
LL	18_46	2.24	H3GA0051040	46.94	0.280	0.551	Intergenic	*NFE2L3* (dist = 690,741)
								*NPVF* (dist = 141,966)
	13_80	2.11	ALGA0071112	80.14	0.288	0.390	Intronic	*MRPS22*
	1_254	1.96	ALGA0009437	254.44	0.309	0.459	Intergenic	*RGS3* (dist = 232,910)
								*ZNF618* (dist = 33,149)
	15_14	1.3	H3GA0043799	14.01	−0.051	0.141	Intergenic	*HNMT* (dist = 244,837)
								*THSD7B* (dist = 75,963)
			ALGA0084000	14.03	0.065	0.170	Intergenic	*HNMT* (dist = 265,141)
								*THSD7B* (dist = 55,659)
			ALGA0083995	14.05	−0.052	0.142	Intergenic	*HNMT* (dist = 286,129)
								*THSD7B* (dist = 34,671)
			ALGA0083978	14.30	−0.015	0.053	Intronic	*THSD7B*
	10_7	1.2	ALGA0056744	7.31	0.161	0.357	Intergenic	*ESRRG* (dist = 289572)
								*GPATCH2* (dist = 295957)
YY	17_13	1.16	ASGA0089601	13.97	0.725	0.843	Intronic	*SLC23A2*

SSC, *Sus Scrofa* chromosome; GV, genetic variance; SNP, single-nucleotide polymorphism.

1) DD, Duroc; LL, Landrace; YY, Yorkshire.

**Table 5:** Summary of informative single-nucleotide polymorphisms in the significant 1-Mb windows for average daily gain by breed

Breed^1)^	SSC_Mb	GV (%)	Informative SNP	Position	Effect	Model frequency	Region annotation	Gene annotation
DD	3_123	3.53	MARC0091117	123.40	−1.122	0.673	Intergenic	*U2* (dist = 481,335)
								*TRIB2* (dist = 426,227)
	17_44	3.52	MARC0028846	44.00	1.430	0.663	Intergenic	*CHD6* (dist = 421,373)
								*PTPRT* (dist = 27,816)
	6_164	2.98	ASGA0098375	164.10	2.335	0.339	Intronic	*MKNK1*
	15_111	2.84	ALGA0086770	111.00	0.672	0.350	Intergenic	*U2* (dist = 1,253)
								*MAP2* (dist = 527,654)
	16_25	2.55	ASGA0072766	25.00	−0.033	0.126	Intergenic	*U2* (dist = 496,722)
								*PTGER4* (dist = 392,964)
			ALGA0089851	25.00	−0.037	0.143	Intergenic	*U2* (dist = 563,131)
								*PTGER4* (dist = 326,555)
			ALGA0089849	25.00	0.047	0.171	Intergenic	*U2* (dist = 529,039)
								*PTGER4* (dist = 360,647)
	13_15	2.11	ALGA0120330	15.00	0.199	0.668	Intergenic	*ZCWPW2* (dist = 312,619)
								*RBMS3* (dist = 472,631)
	1_171	1.4	MARC0001172	171.80	0.067	0.291	Intergenic	*FBXO33* (dist = 1,166,208)
								*U6* (dist = 188,990)
	5_15	1.25	M1GA0007662	15.30	0.065	0.304	Intergenic	*FAIM2* (dist = 26,363)
								*AQP2* (dist = 24,393)
			ASGA0024735	15.30	0.045	0.250	Intronic	*NCKAP5L*
	10_7	1.04	M1GA0013649	7.00	0.010	0.084	Intronic	*GPATCH2*
			DRGA0010240	7.00	0.011	0.074	Intronic	*SPATA17*
			DRGA0010231	7.00	−0.098	0.438	Intergenic	*ESRRG* (dist = 488,896)
								*GPATCH2* (dist = 96,633)
LL	10_11	5.46	DRGA0010301	11.51	0.916	0.9159	Intergenic	*FAM177B* (dist = 17,005)
								*DISP1* (dist = 7,902)
	11_18	1.26	MARC0113984	18.00	0.056	0.0563	Intronic	*RCBTB1*
			CASI0005912	18.11	0.349	0.3486	Intronic	*EBPL, ARL11*
			ALGA0061162	18.12	0.052	0.0518	Intronic	*RCBTB1*
YY	5_95	1.46	ASGA0026863	95.04	0.257	0.992	Intergenic	*C12orf50* (dist = 521,803)
								*MGAT4C* (dist = 1,138,851)
	5_71	1.08	ASGA0026241	71.33	0.011	0.191	Intronic	*SLC2A13*
			ASGA0026236	71.16	0.056	0.338	Intronic	*ABCD2*
			ALGA0032782	71.20	−0.118	0.450	Intronic	*C12orf40*
	1_159	1.08	ALGA0006599	159.66	0.093	0.770	Intergenic	*RNF152* (dist = 58,322)
								*CDH20* (dist = 157,254)
	17_15	1.00	INRA0052808	15.89	0.042	0.493	Intergenic	*BMP2* (dist = 135,631)
								*HAO1* (dist = 847,594)
			INRA0052780	15.65	0.041	0.460	Intergenic	*FERMT1* (dist = 522,008)
								*BMP2* (dist = 90,074)

SNP, single-nucleotide polymorphism; ADG, average daily gain; SSC, *Sus Scrofa* chromosome; GV, genetic variance.

1) DD, Duroc; LL, Landrace; YY, Yorkshire.

**Table 6:** Summary of informative single-nucleotide polymorphisms in the significant 1-Mb windows for 90DAYS by breeds

Breed^1)^	SSC_Mb	GV (%)	Informative SNP	Position	Effect	Model frequency	Region annotation	Gene annotation
DD	6_80	4.86	H3GA0018314	80.70	2.035	0.723	Intronic	*LACTBL1*
	5_15	1.38	M1GA0007662	15.30	−0.048	0.175	Intergenic	*FAIM2* (dist = 26,363)
								*AQP2* (dist = 24,393)
			ASGA0024735	15.30	−0.111	0.382	Intronic	*NCKAP5L*
	16_47	2.38	MARC0030690	47.00	−1.396	0.390	Intergenic	*PIK3R1* (dist = 500,109)
								*SLC30A5* (dist = 261,000)
	16_45	2.16	MARC0002703	45.00	1.228	0.329	Intergenic	*CD180* (dist = 242,747)
								*PIK3R1* (dist = 604,060)
	16_25	2.4	ASGA0072766	25.00	0.040	0.105	Intergenic	*U2* (dist = 496,722)
								*PTGER4* (dist = 392,964)
			ALGA0089851	25.00	0.048	0.126	Intergenic	*U2* (dist = 563,131)
								*PTGER4* (dist = 326,555)
			ALGA0089849	25.00	−0.060	0.153	Intergenic	*U2* (dist = 529,039)
								*PTGER4* (dist = 360,647)
	15_111	1.12	ALGA0086770	111.00	−0.484	0.247	Intergenic	*U2* (dist = 1,253)
								*MAP2* (dist = 527,654)
	12_43	1.77	ALGA0116086	43.00	0.110	0.276	Intronic	*RAB11FIP4*
	1_163	1.09	MARC0004843	163.30	0.016	0.088	Intronic	*IGDCC4*
			ASGA0005079	163.40	0.036	0.148	Intergenic	*IGDCC4* (dist = 7,455)
								*DPP8* (dist = 3,381)
			ALGA0006725	163.80	−0.089	0.311	Intronic	*IGDCC4*
	1_13	1.73	ALGA0001167	13.30	−1.600	0.282	Intergenic	*MYCT1* (dist = 141,875)
								*ESR1* (dist = 386,485)
	10_7	1.07	M1GA0013649	7.00	−0.013	0.075	Intronic	*GPATCH2*
			DRGA0010240	7.00	−0.035	0.144	Intronic	*SPATA17*
			DRGA0010231	7.00	0.109	0.321	Intergenic	*ESRRG* (dist = 488,896)
								*GPATCH2* (dist = 96,633)
LL	10_11	5.07	DRGA0010301	11.51	−0.342	0.861	Intergenic	*FAM177B* (dist = 17,005)
								*DISP1* (dist = 7,902)
YY	5_71	1.54	INRA0019895	71.30	0.121	0.194	Intronic	*SLC2A13*
			ASGA0026241	71.33	−0.023	0.216	Intronic	*SLC2A13*
			ASGA0026236	71.16	−0.142	0.387	Intronic	*ABCD2*
			ALGA0032782	71.20	0.220	0.434	Intronic	*C12orf40*
	5_95	1.5	ASGA0026863	95.04	−0.459	0.990	Intergenic	*C12orf50* (dist = 521,803)
								*MGAT4C* (dist = 1,138,851)
	17_15	1.02	INRA0052808	15.89	−0.062	0.416	Intergenic	*BMP2* (dist = 135,631)
								*HAO1* (dist = 847,594)
			INRA0052780	15.65	−0.089	0.563	Intergenic	*FERMT1* (dist = 522,008)
								*BMP2* (dist = 90,074)

SNP, single-nucleotide polymorphism; SSC, *Sus Scrofa* chromosome; GV, genetic variance; DAYS90, days to 90 kg.

1) DD, Duroc; LL, Landrace; YY, Yorkshire.
